# Preterm births and associated factors among mothers who gave birth in Axum and Adwa Town public hospitals, Northern Ethiopia, 2018

**DOI:** 10.1186/s13104-019-4650-0

**Published:** 2019-10-02

**Authors:** Gebrekiros Aregawi, Nega Assefa, Firehiwot Mesfin, Fissaha Tekulu, Tesfay Adhena, Mussie Mulugeta, Guesh Gebreayezgi

**Affiliations:** 1 Department of Midwifery, College of Medicine and Health Science, Aksum University, Aksum, Ethiopia; 2 School of Nursing and Midwifery, College of Health and Medical Sciences, Haramaya University, P. O. Box 235, Harar, Ethiopia; 3 Department of Midwifery, College of Medicine and Health Sciences, Arba Minch University, Arba Minch, Ethiopia; 4 Department of Midwifery, College of Health Science, Aksum University, Aksum, Ethiopia; 5 Department of Public Health, College of Health Science, Aksum University, Aksum, Ethiopia

**Keywords:** Preterm birth, Prevalence, Risk factors, Axum and Adwa, Ethiopia

## Abstract

**Objective:**

The objective of this study was to determine the prevalence and associated factors of preterm births among mothers who gave birth in Axum and Adwa public hospitals, Tigray, North Ethiopia, 2018.

**Result:**

This study showed that 13.3% from the total 472 mothers gave a preterm birth. Being a rural resident (AOR = 2.13, 95% CI (1.07,4.22), short inter pregnancy interval (AOR = 5.4, 95% CI (1.32, 22.05), previous preterm birth (AOR = 3.74, 95% CI (1.03, 16.34), Premature rupture of membrane (AOR = 4.14, 95% CI (1.92, 8.89), induced onset of labor (AOR = 2.49, 95% CI (1.06, 5.85) multiple pregnancy (AOR = 5.69, 95% CI (2.27, 14.28), malaria during pregnancy (AOR = 4.71, 95% CI (1.98, 11.23), Presence of chronic illness (AOR = 4.55, 95% CI (1.83, 11.26) were significantly associated with preterm birth.

## Introduction

Preterm birth (PTB) refers to the birth of a baby that occurs before 37 completed weeks of gestation [1]. The complications of preterm birth are the single largest direct cause of neonatal deaths, responsible for 35% of the world’s 3.1 million deaths a year [2].

Preterm birth has many long and short term consequences like cerebral palsy, mental retardation, visual and hearing impairments, learning difficulties, poor health and growth [3, 4].

Globally WHO estimates of global rates of preterm births indicate that of the 135 million live births in 2010, 15 million babies were born too early, representing a preterm birth rate of 11.1% and over 60% of preterm births occurred in sub-Saharan Africa and South Asia [5].

About 350,000 under five children die annually due to the complication of preterm birth in Africa including Ethiopia. And the highest rates of preterm morality are in West Africa, particularly in the countries currently being decimated by Ebola, notably Sierra Leone and Liberia [6].

In the previous studies, previous history of preterm birth was the strongest predictor of risk for subsequent preterm delivery and if these risk factors are not correctly identified and adequately managed, they contribute to the increasing incidence of preterm births. So, Identification and timely referral for further evaluation and management of these risky women during early pregnancy, is important in decreasing the consequences of preterm birth [7].

Developing countries like Ethiopia lacks reliable data on preterm birth depending largely on estimates from delivery records but the records lack consistency and completeness. Despite tremendous improvement in newborn care, prevention of preterm birth has remained largely unaddressed. Therefore, this study has gone a long way in providing relevant data regarding the factors associated with preterm birth among women gave birth in Axum and Adwa Town public hospitals.

## Main text

### Study design and setting

Institutional based cross-sectional study was conducted in Axum and Adwa town public hospitals from February 08-March 08, 2017. There are four health centers, two general hospitals and one referral hospital in Axum and Adwa town and all are governmental hospitals.

### Sample size and Sampling procedure

The sample size was determined using single population proportion formula by considering confidence level (95%), margin of error = 0.04 and 11.6% of prevalence taken from the study done in Debre Markos [8]. By adding 10% non-response rate, the final sample size was 482. Mothers who gave birth in Axum and Adwa town public hospitals with known LNMP or had ultrasound result during the first trimester of pregnancy were included. Sample sizes were proportionally allocated to each hospital. By using Systematic random sampling method every second mother was recruited into the study.

### Data collection tools and techniques

Face-to-face interviewer administered questionnaires and mother’s profile card was used to collect the data from postpartum mothers and gestational age was determined by last normal menstrual period and by early ultrasound check up result.

### Data quality control

Questionnaire was pretested on 5% of the sample size in suhul shire hospital to ensure its completeness. Data entry was done by two data clerks. Finally, multivariate analysis was run in the binary logistic regression model to control the confounding factors.

### Data processing and analysis

Data was entered in Epi Data 3.1 and exported into SPSS version 22 for analysis. Both bivariable and multivariable logistic regression analysis were carried out to identify factors associated with preterm birth. In bivariate logistic regression analysis, variables with *p* value less than 20% were considered into the multivariable analysis. Adjusted odds ratio with a 95% confidence interval was calculated to see the strength and significant association. Variables having a p-value less than 0.05 in the multivariable logistic regression analysis were considered as significant.

## Results

### Socio-demographic characteristics of the respondents

A total of 472 mothers were included in this study with a response rate of 97.9%. The median age of the study participants was 26.00 with interquartile range of ± 8. 75.6% of the study participants were between 20 and 34 years old. Concerning to maternal level of education status of the respondents, 72.8% of the mothers were attended a formal education (Table 1).

**Table 1:** Socio-demographic characteristics of women who give birth in Axum and Adwa town public hospitals, Tigray, Northern Ethiopia, February 08–March 08, 2018

Variables	Frequency	Percentage
Maternal age		
≤19	42	8.9
20–34	357	75.6
≥35	73	15.5
Maternal level of education (n = 472)		
Unable to read and write	108	22.9
Able to read and write	10	4.2
Primary level	103	21.8
Secondary level	161	34.1
College and above	80	16.9
Maternal marital status (n = 472)		
Single	11	2.3
Married	446	94.6
Divorced	11	2.3
Widowed	4	0.8
Maternal occupation (n = 472)		
Housewife	265	56.1
Private employee	87	18.4
Government employee	84	17.8
Merchant	30	6.4
Student	6	1.3
Maternal religion (n = 472)		
Orthodox	435	92.2
Muslim	32	6.8
Protestant	3	0.6
Catholic	2	0.4
Ethnicity		
Tigray	459	97.2
Amara	11	2.3
Others*	2	0.4
Residence (n = 472)		
Urban	308	65.3
Rural	164	34.7

Asterisk represent the list of other ethinicities like Oromo, Wolayta, Guragge other than Tigray and Amara

### Obstetrics related information of the mothers

Out of 472 mothers, 264 (55.9%) of them were multipara and the rest 208 (44.1%) mothers were prim Para. Regarding the mode of delivery for recent pregnancy, 367 (77.8%) out of 472 mothers were gave birth via spontaneous vaginal delivery. the rest 32 (6.8%) and 73 (15.5%) of mothers gave birth via assistance of instrumental and caesarean section, respectively (Table 2).

**Table 2:** Obstetrics related information of mothers who gave birth in Axum and Adwa town public hospitals, Tigray, North Ethiopia, and February 08–March 08, 2018

Variables	Frequency	Percentage
Parity (n = 472)		
Multipara	264	55.9
Primipara	208	44.1
Number of parity		
1	39	14.8
2–4	187	70.8
≥5	38	14.4
Inter pregnancy interval (n = 264)		
<24 months	38	14.4
>24 months	226	85.6
Previous preterm birth (n = 264)		
Yes	34	12.9
No	230	87.1
History of abortion (n = 472)		
Yes	71	15
No	401	85
Pregnancy status (n = 472)		
Planned	434	91.9
Unplanned	38	8.1
Onset of labour (n = 472)		
Spontaneous	405	85.8
Induced	67	14.2
Mode of delivery		
Vaginal delivery	367	77.8
Instrumental	32	6.8
Caesarean section	73	15.4
PROM (n = 472)		
Yes	68	14.4
No	404	85.6
History of PIH (n = 472)		
Yes	38	8.1
No	434	91.9
History of APH (n = 472)		
Yes	39	8.3
No	433	91.7
Pregnancy type (n = 472)		
Singleton	434	91.9
Multiple	38	8.1

### Health care service use characteristics

From 472 mothers, 452 (95.8%) had ANC follow up for the recent pregnancy. Among these mothers who had ANC follow up about 325 (71.9%) of interviewed mothers had four or more visits.

### Medical related information of the mothers

Out of 472 interviewed mothers, 461 (97.7%) were tested for HIV and 449 (97.4%) of them were negative. Concerning to the mothers haemoglobin level check-up, 462 (97.9%) of them were checked for their haemoglobin level (Additional file 1: Table S1).

### Prevalence of preterm birth

Out of 472 mothers, 63 (13.3%) mothers gave preterm babies. These preterm babies were diagnosed by calculating the gestational age from the last normal menstrual period/using early ultrasound result.

### Factors associated with preterm births

In this study, Mothers who were living in rural area were two times more likely [(AOR = 2.1 (1.05, 4.12)] to have preterm birth compared to those who live in urban area. Concerning to inter pregnancy interval, mothers with inter pregnancy interval less than 24 months four [(AOR = 4.24, 95% CI (1.15, 16.26)] times were more likely to give preterm births compared to mothers who had inter pregnancy interval greater. Regarding the previous history of preterm birth, mothers who had history of preterm births were four [(AOR = 3.74, 95% CI (1.03, 16.34)] times more likely to give preterm birth compared to mothers who had no previous preterm births.

Coming to the obstetric complications during the pregnancy like PROM, mothers who encountered PROM [(AOR = 3.76 (1.73, 8.19)] during the pregnancy were almost four times more likely to have preterm birth compared to mothers who had no PROM. Mothers with multiple pregnancy outcomes [(AOR = 5.59 (2.17, 14.40)] were six times more likely to have preterm birth compared with mothers who gave birth singleton.

Concerning to the medical factors, mothers who had exposed for malaria during pregnancy [(AOR = 5.43 (2.19, 13.38)] and chronic medical disorders [(AOR = 6.79 (2.83, 16.26)] were seven times more likely to have preterm births than the counterpart (Additional file 2: Table S2).

## Discussion

In this study, the prevalence of preterm births in this study was found to be 13.3%.

The result of this study was consistent with the study done in Debre Markos (2013) and India (2010) which reported with the prevalence of 11.6% and 15% [8, 9]. This similarity might be due to health care system in our country and service provided for mothers are almost uniform through out the different area of the country.

The finding of this study was higher than the study conducted in Gondar (2012), Iran Asali Hospital (2007) and Egypt (2014) which reported that the prevalence of preterm birth was 4.4% 6.3% and 8.2% [10–12], respectively. This discrepancy might be due to difference in inclusion and exclusion criteria, study areas and due to difference in health care services provided.

The finding of this study was found to be lower than studies conducted in Nigeria (2010) with the prevalence rate of 24% [13]. This might be due to the higher rate of multiple gestations in Nigeria, as this cause over distended uterus and can precipitate to preterm birth and multiple gestations is a known predisposing factor for preterm birth.

The finding in this study was also lower than the study done in Kenya (2013), Brazil (2009) and India (2010) which reported that the prevalence were 18.3% and 21.7% [14, 15]. These discrepancies might be due to difference in study areas, methodological differences.

This study showed that residence was associated significantly with preterm birth. This finding was in line with the study done in Bahirdar [16]. This might be due low access of maternal care services and awareness differences of the mothers in the rural area with mother found in the urban area.

Short inter pregnancy (< 24 months) was significantly associated with preterm birth. This finding was in consistence with the study conducted in Debre Markos since 2013 [8]. This is a risk factor for preterm birth and it might be due to existence of unidentified factors which precipitating preterm births in mothers with short inter pregnancy interval.

Previous preterm birth was another factor significantly associated with preterm birth found in this study. Mothers who had previous history of preterm births seven times were more likely to have preterm births compared to mothers who had no previous history of preterm births in the subsequent deliveries. This finding was in consistence with the secondary data analysis report of Malawi (2014): [17]. The possible reason might be due to existence of unidentified factors which precipitating preterm births in the subsequent deliveries.

According to this study, mothers who had premature rupture of membranes (PROM) during pregnancy were four times more likely to have preterm birth compared to those had no premature rupture of membrane. This finding was in consistence with findings of cross sectional studies conducted in Debre Markos (2013) and Kenya (2013) [8, 15]. This may be due to the fact that PROM elevated fetal plasma interleukin-6 indicating that this fetal response may trigger preterm labor correlated strongly with spontaneous delivery [18].

Multiple gestations were significantly associated with preterm birth in this study. Mothers with multiple pregnancies were almost six times more likely to have preterm birth compared with mothers who gave birth singleton This finding was in line with the finding of study done in Kenya (2013) [15]. This might be due to multiple gestation is associated with uterine over distension and this may result in spontaneous preterm labour and also Multiple pregnancies can stretch the myometrium; this induces the oxytocin receptors, which results in preterm labor and delivery [19].

Chronic medical illnesses are significant variable associated with Preterm birth. Mothers with chronic medical disorders were almost seven times more likely to have preterm births compared to mothers who had not chronic medical disorders. This finding was in consistence with cross sectional study conducted in Debre Markos (2013) [8]. This might be might be due to maternal illnesses can limit or minimize the placental delivery of oxygen and nutrients to the developing fetus in the uterus possibly increase the risk of preeclampsia and, thus, increases the risk preterm birth [20].

Besides to this, mothers who had malaria during their pregnancy were more and mothers who had exposed for malaria during pregnancy were five times were more likely to have preterm births than mothers who had not exposed to malaria during the pregnancy. This result was in line with the study done in Iran (2011–2013) [21]. This might be due to malaria affects placenta and contributes to maternal anaemia and placental parasitemia which leads to preterm labor which in turn result preterm birth [22].

### Conclusion

Residence, short inter pregnancy, previous history of preterm birth, PROM, induced onset of labor, multiple gestation, Presence of chronic medical illness, being exposed for malaria during the pregnancy were statistically significant with preterm birth. Therefore, efforts have to be made to decrease the magnitude of preterm births and further improvements of antenatal care as well as early screening are important recommendations.

## Limitation of the study

Being a cross-sectional study design attempt to our work did not establish the possible temporal relationship between dependent and independent variables. Besides, recall bias in finding out the gestational age of pregnant women was not ruled out.

## Supplementary information

**Additional file 1: Table S1.** Medical related information of women who give birth in Axum and Adwa town public hospitals, Tigray, Northern Ethiopia, February 08–March 08, 2018.

**Additional file 2: Table S2.** Factors associated with preterm births among mothers who gave birth in Axum and Adwa town public hospitals, Tigray, North Ethiopia, February 08–March 08, 2018.

## Data Availability

The datasets used and/or analysed during the current study are available from the corresponding author upon reasonable request due to ethical restriction and privacy.

## Abbreviations

ANC: antenatal care
AOR: adjusted odd ratio
APH: ante partum hemorrhage
CI: confidence interval
DM: diabetes mellitus
HIV: human immune deficiency virus
PIH: pregnancy induced hypertension
LNMP: last normal menstrual Period
COR: crude odds ratio
PROM: premature rupture of membrane
SPSS: Statistical Package for Social Sciences
