# Supplementary material for: Preterm births and associated factors among mothers who gave birth in Axum and Adwa Town public hospitals, Northern Ethiopia, 2018
Source: BMC Res Notes. 2019 Oct 2;12:640. doi: 10.1186/s13104-019-4650-0 (PMC6775657; doi:10.1186/s13104-019-4650-0)
Supplement: Supplementary file 1 — Additional file 1: Table S1. Medical related information of women who give birth in Axum and Adwa town public hospitals, Tigray, Northern Ethiopia, February 08–March 08, 2018. [file 13104_2019_4650_MOESM1_ESM.docx]

Table S1: Medical related information of women who give birth in Axum and Adwa town public hospitals, Tigray, Northern Ethiopia, February 08-March 08, 2018.

| **Variable** |  | **Frequency** | **Percentage** |
| --- | --- | --- | --- |
| HIV tested (n=472) | Yes  No | 461  11 | 97.7  2.3 |
| HIV status (n=461) | Positive | 13 | 2.8 |
|  | Negative | 448 | 97.2 |
| Haemoglobin checked (n=472**)** | Yes  No | 462  10 | 97.9  2.1 |
| Haemoglobin level(n=462) | <11g/dl  >=11g/dl | 82  380 | 17.7  82.3 |
| UTI during pregnancy (n=472) | Yes | 33 | 7.0 |
|  | No | 439 | 93.0 |
| Malaria positive test during pregnancy (n=472) | Yes | 38 | 8.1 |
|  | No | 434 | 91.9 |
| History of medical chronic illnesses(n=472) | Yes  No | 42  430 | 8.9  91.1 |
| Un prescribed Medication during  Pregnancy(n=472) | Yes  No | 6  466 | 1.3  98.7 |
