# Supplementary material for: Preterm births and associated factors among mothers who gave birth in Axum and Adwa Town public hospitals, Northern Ethiopia, 2018
Source: BMC Res Notes. 2019 Oct 2;12:640. doi: 10.1186/s13104-019-4650-0 (PMC6775657; doi:10.1186/s13104-019-4650-0)
Supplement: Supplementary file 2 — Additional file 2: Table S2. Factors associated with preterm births among mothers who gave birth in Axum and Adwa town public hospitals, Tigray, North Ethiopia, February 08–March 08, 2018. [file 13104_2019_4650_MOESM2_ESM.docx]

#### Table S2: Factors associated with preterm births among mothers who gave birth in Axum and Adwa town public hospitals, Tigray, North Ethiopia, February 08-March 08, 2018.

| Independent variables | Frequency (%) | Preterm birth | | COR (95% CI) | AOR (95% CI) |
| --- | --- | --- | --- | --- | --- |
|  |  | Yes | No |  |  |
| Residence |  |  |  |  |  |
| Urban | 308 (65.3) | 29 | 279 | 1.00 | 1.00 |
| Rural | 164 (34.7) | 34 | 130 | 2.52 (1.47, 4.31) * | 2.1 (1.05, 4.12)* |
| Inter pregnancy interval |  |  |  |  |  |
| <24 months | 38 (14.4) | 18 | 20 | 8.78(4.01, 19.06)** | 4.24 (1.15, 16.26)* |
| >=24 months | 226 (85.6) | 21 | 205 | 1.00 | 1.00 |
| Previous preterm birth |  |  |  |  |  |
| Yes | 34 (12.9) | 15 | 19 | 6.78 (3.05, 15.05)** | 6.89 (2.04, 23.32)* |
| No | 230 (87.1) | 24 | 206 | 1.00 | 1.00 |
| Abortion history  Yes  No | 71(15)  401(85) | 15  48 | 56  353 | 1.97(1.03, 7.75) *  1.00 | 1.17(.49, 2.83)  1.00 |
| PROM |  |  |  |  |  |
| Yes | 68 (14.4) | 24 | 44 | 5.10(2.81, 9.27) ** | 3.76(1.73, 8.19) ** |
| No | 404(85.6) | 39 | 365 | 1.00 | 1.00 |
| Onset of labour  Spontaneous  Induced | 405(85.8)  67(14.2) | 40  23 | 365  44 | 1.00  4.78(2.62, 8.69) ** | 1.00  2.49(1.06, 5.85) * |
| APH |  |  |  |  |  |
| Yes | 39 (8.3) | 13 | 26 | 3.83 (1.85, 7.93) ** | 1.96(0.66, 5.80) |
| No | 433 (91.7) | 50 | 383 | 1.00 | 1.00 |
| PIH |  |  |  |  |  |
| Yes | 38 (8.1) | 15 | 23 | 5.24(2.56, 10.74)** | 2. 59 (0.99, 6.77) |
| No | 434(91.9) | 48 | 386 | 1.00 | 1.00 |
| Pregnancy type |  |  |  |  |  |
| Singleton  Multiple | 434 (91.9)  38(8.1) | 46  17 | 388  21 | 1.00  6.83 (3.36, 13.87)** | 1.00  5.59 (2.17, 14.40) ** |
| Hemoglobin level  >=11 g/dl  <11g/dl | 380(82.3)  82(17.7) | 39  23 | 341  59 | 1.00  3.41(1.89, 6.12) ** | 1.00  1.31(0.58, 2.93) |
| Malaria positive test during pregnancy |  |  |  |  |  |
| Yes | 40 (8.5) | 16 | 22 | 5.98 (2.94, 12.20)** | 5.43 (2.19, 13.38) ** |
| No | 432 (91.5) | 46 | 386 | 1.00 | 1.00 |
| Chronic medical illnesses |  |  |  |  |  |
| Yes | 42 (8.9) | 21 | 21 | 9.24 (4.66, 18.29) ** | 6.79 (2.83, 16.26)* |
| No | 430 (91.1) | 42 | 388 | 1.00 | 1.00 |
| **Key**: *=p-value <0.05, **=p value<0.001, CI = Confidence Interval, COR = Crude Odds Ratio, AOR = Adjusted Odds Ratio | | | | | |
